# Binary Effect of Fly Ash and Palm Oil Fuel Ash on Heat of Hydration Aerated Concrete

**DOI:** 10.1155/2014/461241

**Published:** 2014-02-13

**Authors:** Taha Mehmannavaz, Mohammad Ismail, Salihuddin Radin Sumadi, Muhammad Aamer Rafique Bhutta, Mostafa Samadi, Seyed Mahdi Sajjadi

**Affiliations:** Construction Research Centre, Faculty of Civil Engineering, Universiti Teknologi Malaysia, 81310 Johor Bahru, Johor, Malaysia

## Abstract

The binary effect of pulverized fuel ash (PFA) and palm oil fuel ash (POFA) on heat of hydration of aerated concrete was studied. Three aerated concrete mixes were prepared, namely, concrete containing 100% ordinary Portland cement (control sample or Type I), binary concrete made from 50% POFA (Type II), and ternary concrete containing 30% POFA and 20% PFA (Type III). It is found that the temperature increases due to heat of hydration through all the concrete specimens especially in the control sample. However, the total temperature rises caused by the heat of hydration through both of the new binary and ternary concrete were significantly lower than the control sample. The obtained results reveal that the replacement of Portland cement with binary and ternary materials is beneficial, particularly for mass concrete where thermal cracking due to extreme heat rise is of great concern.

## 1. Introduction 

The hydration of cement compounds is exothermic where heat is liberated to the surrounding. In other words, when cement is hydrated, the compounds react with water to acquire stable low-energy states, and the process is accompanied by the release of energy in the form of heat [1]. The quantity of heat developed upon complete hydration of a certain amount of unhydrated cement at a given temperature is defined as heat of hydration [2]. The significance of heat of hydration in concrete technology is manifold. The total amount of heat liberated and the rates of heat liberation from hydration of the individual compounds can be used as indices of their reactivity [3]. Furthermore, heat of hydration describes the setting and hardening behaviour of cement and predicts the temperature rise as well. The temperature of concrete due to hydration is largely controlled by materials and mix properties and by environmental factors [3]. In fact, the heat of hydration depends on the chemical behaviour of the compounds and is nearly equal to the sum of the heats of hydration of the individual pure compounds when their respective proportions by mass are hydrated separately. Again the major constituent of portland cement is lime inform of tricalcium and di-calcium. The triclacium phase is responsible for early strength development and di-calcium for strength development after 28 days of hydration. Therefore the development of total heat will surely be affected by the quantity of cement in the mix. High cement contents may be beneficial to obtain higher initial strengths in concrete, but the greater heat developed due to the chemical reactions produces unwanted cracks and shrinkage in the concrete [4, 5].

Regarding the durability of concrete structures, modern concrete practices stipulate special measures to reduce peak and differential temperatures using materials with lower release of heat in order to minimise or prevent thermal cracking thereby avoiding the corrosion of embedded steel reinforcement [1, 6]. Like that of chemical admixture, the use of pozzolanic materials in reducing heat of hydration in concrete is well established [1, 3, 7]. Unlike OPC, pozzolan starts reacting somewhat belatedly with the calcium hydroxide produced by clinker hydration and therefore, at least initially, it behaves like an inert diluting agent towards the Portland cement with which it has been mixed. These phenomena result in a reduced rate of heat evolution, that is, rise in temperature, and reduce ultimate heat of hydration. In this regard, the use of fly ash as a conventional replacement in reducing heat of hydration of concrete is well established. Perhaps the first field trials with the use of fly ash were made in 1950 at the Otto Holden Dam on the Ottawa River near Mattawa, Ontario [8]. It has been found that concrete containing 30% fly ash lowered the maximum temperature rise by 30 percent. The inclusion of higher amount (equal or over 50%) of fly ash in concrete has been shown to be beneficial in many aspects of durability including thermal cracking due to heat liberation [9]. Other pozzolanic materials like slag, silica fume, rice husk ash, and so forth have been shown to influence the concrete by lowering the adiabatic heat in concrete mass [8, 10–13].

Effort towards sustainability and sustainable environment as made possible the use of pozzolanic material in concrete and other construction related materials. One of the latest additions of pozzolanic material is palm oil fuel ash (POFA). A waste material obtained from burning of palm oil husk, shell and fibre as fuel for palm oil mill boilers that has been identified as a good pozzolanic material [14–16]. This waste is commonly available in South-East Asia and African sub-Sahara region where palm oil production plays an important role in the national economy. It has been estimated that the total solid waste generated by the industry in Malaysia, alone has amounted to about ten million tons a year [17]. In view of the utilization of POFA as a supplementary cementing material, extensive research works have been carried out in the Faculty of Civil Engineering, Universiti Teknologi Malaysia in examining various aspects of fresh and hardened state properties of concrete. This paper presents experimental results on the effect of palm oil fuel ash in reducing heat of hydration of concrete.

## 2. Materials and Test Methods 

The name of materials that are used through this research and their characteristics are listed in this section.

*Cement.* Ordinary portland cement (OPC) was prepared from the Tasek Corporation Berhad-Cement Industry of Malaysia. The OPC used complies with the Type I of the portland cement as stated in the ASTM C 150-05 [18].

*POFA.* Palm oil fuel ash (POFA) used was a by-product obtained from burning of palm oil shell and husk at temperature of 940°C from a Kahang mill, Kluang Johor, southern state of Malaysia. Afterwards, as suggested by Hussin and Awal [19], the POFA was grounded by using a modified Los Angeles abrasion test machine. The machine had 8 stainless bars, each of which is 12 mm in diameter and 800 mm long, in order to accurately produce extremely fine particles. Then, the collected POFA was dried in the oven at the temperature of 110°C ± 5°C for 24 h to remove moisture in it before sieved and ground to obtain finer particles.

*Pulverized Fuel Ash (PFA).* Pulverized fuel Ash (PFA) used for this research was obtained from the silos of Kapar Power Station, located Selangor, Malaysia. The fineness of PFA complies with the specifications of ASTM C 618-05 (2005).

*Fine Aggregate.* In the case of preparing all the aerated concrete specimens, the aggregate used only comprises bottom Ash from Tanjung Bin power plant in Pontian, Johor. The Bottom ash is transported from the bottom of the boiler to the ash pond as liquid slurry by 200–250 mm diameter pipes. Initially the bottom ash was dried in an oven at the temperature of 105°C ± 5°C for 24 hours. Then, the oven-dried bottom ash is sieved passing 1.18 mm sieve before stored in the airtight container.

*Water*. In this experiment, tap water was used for the manufacture of the lightweight concrete.

*Superplasticizer*. The trade name of the superplasticizer used during this study was SIKAMENT NN. This superplasticizer was used as a chemical admixture. According to ASTM C494/C494 M-05 [20] that is known as a type F which is a high range water reducing admixture. The used superplasticizer was from group sulphonated naphthalene formaldehyde condensates (SNF) and it was dry powder form.

*Aluminum Powder*. The type of the aluminum powder used through this study was Y250. It was the gaseous agent producing the porosity within the mass of aerated concrete.

*Thermocouple*. Thermocouple used in this investigation was VITAR heater and thermosensor from the Vitar Group Heat and Temperature Division, Malaysia.

### 2.1. Physical and Chemical Composition of POFA and PFA

**Figure 1:**
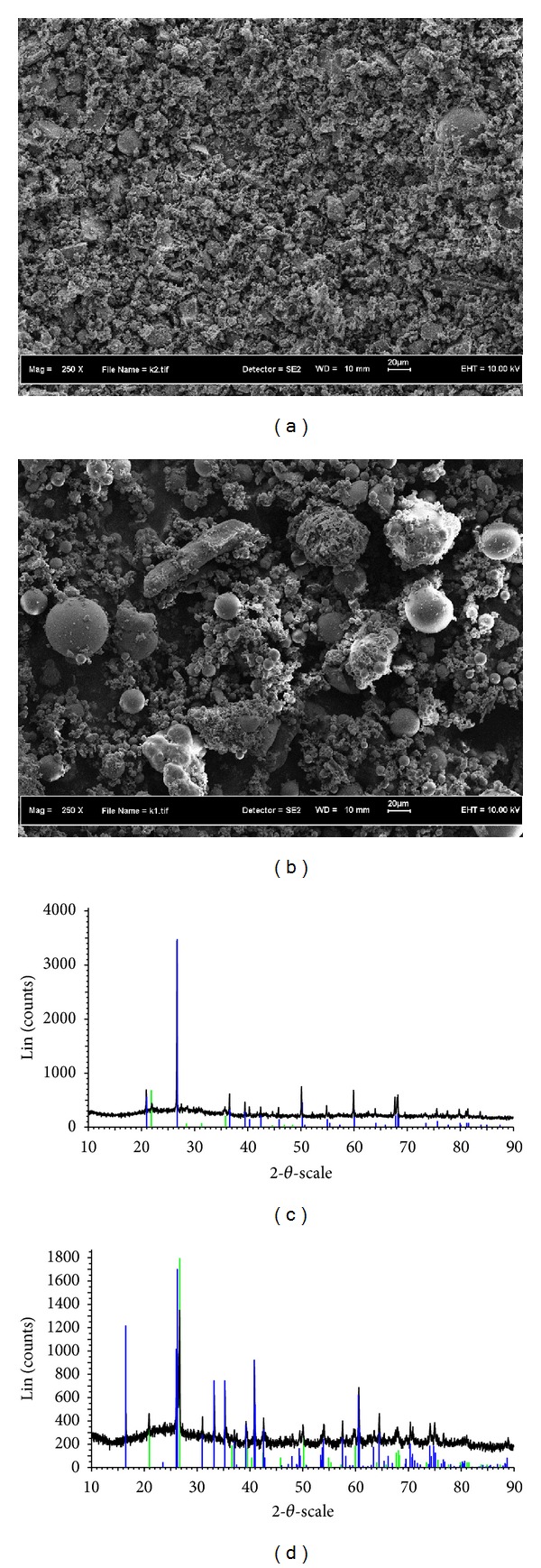
(a) Scanning electron micrograph of POFA, (b) scanning electron micrograph of PFA, (c) X-ray diffraction pattern POFA, and (d) X-ray diffraction pattern PFA.

The chemical composition and physical properties of OPC, POFA, and PFA were shown in Tables 1 and 2, respectively. From the Table 1 it can be seen that the materials (POFA and PFA) contain high silica oxide which influences pozzolanic reaction when it reacts with free lime. Thereby, creating extra calcium silicate hydrate (C-S-H) gels, which has merit to strength development of the POFA and PFA concrete. The sum of silica, aluminium, and iron oxide of POFA and PFA is greater than 75% of the total chemical composition, which makes this pozzolanic material be classified between class C and F pozzolan [21]. However, the material contains low calcium oxide (CaO), that is, in the range of 4–8%, and low silicate (SO_2_) within the minimum allowable that was mentioned by the ASTM C 618-12A [21]. The materials are relatively spherical, irregular particles in shape. A typical electron micrograph and X-ray diffraction pattern of the ashes are shown in Figures 1(a)–1(d).

### 2.2. Aerated Concrete Mixes

In the present investigation three types of the aerated concrete were made; first type (I) was with the OPC alone, used as a control sample, In the second type (Type II) the OPC was replaced by weight of 50% POFA, and in the third type (Type III), the OPC was replaced by weight of 30% POFA and 20% PFA. The mixtures (i.e., 50% POFA in the Type II, and that of 30% and 20% POFA and PFA in Type III) were selected according to the literatures that aimed only to improve the compressive strength of concrete [22–26]. The detail of the mix proportions of three types of aerated concrete was shown in Table 3.

## 3. Methodology 

**Figure 2:**
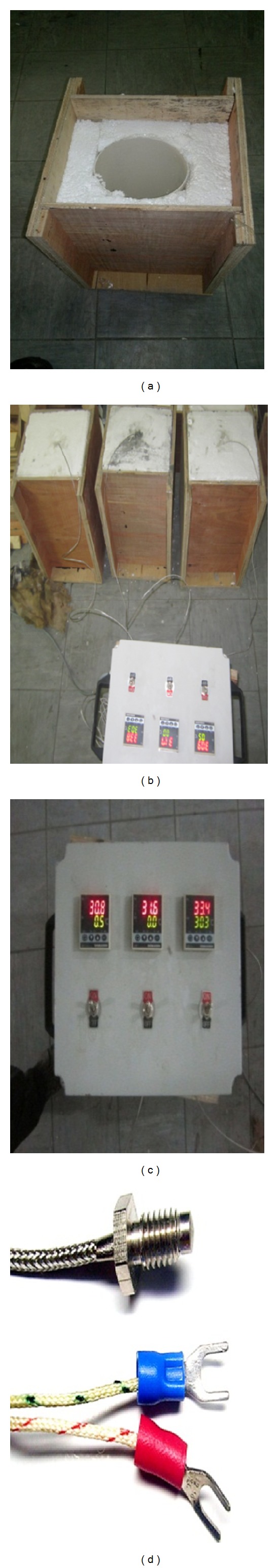
Internal view of an insulated plywood box and test arrangement and test instrument.

A cubical plywood with the size of 280 mm was used as the exterior mould. Then, after installing the interior concrete mould, it was internally packed with 76 mm thick expanded polystyrene acting as the insulator (Figure 2(a)). Each aerated concrete mix was cast into another PVC pipe with size of 150 mm diameter and 300 mm height. A general view of the plywood boxes and the test arrangement is shown in Figure 2(b). Prior to casting, a thermocouple (Type K) was inserted into the centre of each box through the drilled hole of the polystyrene foam lid and was connected to data acquisition (data logger) system (Figures 2(b) and 2(c)).  Figure 2(d) also showed the thermocouple wire that is used into the aerated concrete in order to measure the head of hydration during the test process.  Table 4 presented the characteristic of Thermocouple Type K.

When aerated concrete was poured into the box, heat was liberated by the hydration process that subsequently increased the temperature of the aerated concrete mass. This increase in temperature and subsequent drop was monitored with close intervals during the first 24 hours. However measuring the heat of hydration was later monitored with lesser frequents until the temperature dropped close to its initial reading (first measurement when the test is just started). Recording the temperature was continued up to 5 days for the whole three aerated concrete types.

## 4. Results and Discussion

**Figure 3:**
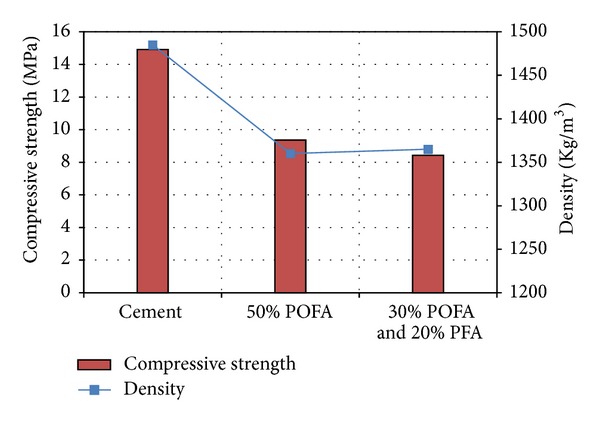
Relationships between compressive strength and density for aerated concrete (28 days).

**Figure 4:**
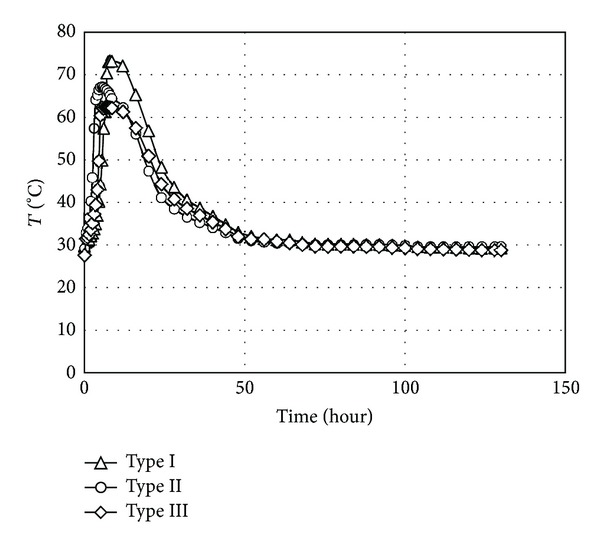
Development of heat of hydration.

The results of variation of the temperature versus time were recorded for the different aerated concrete types and presented in Table 5. In addition, the relationship between compressive strength and the density for all three aerated concrete types is showed in Figure 3. The development of temperature (i.e., due to heat liberation) was also obtained in the mid depth of aerated concrete during hydration process for the all aerated concrete samples and the results are showed in Figure 4. It has been observed that during the initial stage, the temperature rise due to heat of hydration of all the specimens was approximately equal. However, as testing time increased the influence of ash replacement on heat of hydration was observed. The specimens containing POFA and PFA demonstrated lower heat rise as compared to the controlled sample (Type I). Type II and Type III of the aerated concrete could successfully reduce the total temperature rise compared to the Type I. Also, the time at which the peak temperatures occurred increased compared to the Type I aerated concrete (Figure 4).

Although the initial temperature of the whole aerated concrete types was approximately same, more heat was considerably obtained from Type I aerated concrete during the first day of experiment, particularly in the first 8 hours after casting. A peak temperatures of 73.3°C was observed for Type I aerated concrete at 8 hours, while the peak temperature of 67.2 and 62.3°C were observed for Type II and Type III aerated concrete at 5: 30 hours, respectively (Figure 4).

It is indeed well established that the fineness of cement influences the rate of heat development to some extent [1]. In spite of the higher amount of POFA and PFA, no adverse effect was detected on the fineness of the cement. The peak temperatures obtained for neither Type II nor Type III aerated concrete were recorded to be higher than Type I. In case of the heat of hydration reduction, both Type I and Type II performed more effective than the Type I, especially in the early steps where the peak in the temperature was measured. This was also reported by Sata et al. [27] for the normal concrete mixed with the POFA. They have monitored a reduction of 15% temperature in concrete incorporating with the 30% POFA comparing to the concrete with OPC alone.

## 5. Conclusions 

POFA is relatively a new pozzolanic material and until now it does not have any specification compared to PFA. It has been characterized to be a unique supplementary cementing material for the concrete. The obtained results in this study demonstrate that the partial replacement of cement by POFA and PFA is advantageous and has very good potential to control the heat of hydration in concrete particularly in the first days of concrete casting. It can be concluded that is by incorporating 50% ashes (i.e., the aerated concrete, namely, Type II and Type III made for this study) as a replacement to OPC (Type I aerated concrete), the heat of hydration significantly reduced through the aerated concrete. However there is limit on the usage of ashes because higher amount of ashes can cause strength reduction in the aerated concrete. To design an appropriate mix proportion for the aerated concrete, particularly in places that the thermal cracking (due to excessive heat rise) is of great concern, exploring the optimized amount of ashes that satisfy both the strength and heat of hydration factors is a key matter.

## Figures and Tables

**Table 1:** Chemical composition of OPC, POFA, and PFA.

Chemical combination (%)	OPC	POFA	PFA
SiO_2_	16.40%	63.70%	53.60%
Al_2_O_3_	4.24%	3.68%	26.60%
Fe_2_O_3_	3.53%	6.27%	5.36%
CaO	68.30%	5.97%	7.28%
K_2_O	0.22%	9.15%	1.30%
P_2_O_5_	—	4.26%	1.51%
MgO	2.39%	4.11%	0.67%
CO_2_	0.10%	0.10%	0.10%
SO_3_	4.39%	1.59%	0.63%
Cl	—	0.50%	—
TiO_2_	—	0.30%	1.94%
Mn	—	0 < LLD	0 < LLD
Na	—	0 < LLD	—
Mno	0.15%	—	—
Ti	0 < LLD	—	—
SrO	—	—	0.33%
BaO	—	—	0.20%
Zr	—	—	0 < LLD

**Table 2:** Physical properties of OPC, POFA, and PFA.

Physical properties	OPC	POFA	PFA
Specific gravity	3.15	2.42	2.62
Particle retained on 45 µm sieve	4.58	4.98	6.92
Median particle d10	—	1.69	—
Median particle d50	—	14.58	—
Blaine fineness (cm^3^/g)	3999	4935	3205
Soundness (mm)	1.0	2.0	—
Strength Activity Index (%)			
At 7 days	—	80	84
At 28 days	—	84	92

**Table 3:** Mix proportions of OPC and POFA-PFA aerated concrete.

Materials	OPC	50% POFA	30% POFA and 20% PFA
OPC (kg/m)	600	300	300
POFA (kg/m^3^)	—	300	180
PFA (kg/m^3^)	—	—	120
Bottom ash (kg/m^3^)	600	600	600
Water (kg/m^3^)	276	276	276
Aluminum powder	3.6	3.6	3.6
Superplasticizer	2.4	2.4	2.4

**Table 4:** Characteristic of thermocouple Type K.

Type	Temperature range °C (continuous)	Temperature range °C (short term)	Tolerance class one (°C)	Tolerance class two (°C)
K	0 to +1100	−180 to +1300	±1.5 between −40°C and 375°C ±0.004 × *T* between 375°C and 1000°C	±2.5 between −40°C and 333°C ±0.0075 × *T* between 333°C and 1200°C

**Table 5:** Characteristic of heat of hydration of OPC and POFA-PFA aerated concrete.

Properties	OPC	50% POFA	30% POFA and 20% PFA
Initial temperature (°C)	29.7	29.2	27.6
Peak temperature (°C)	73.3	67.2	62.3
Time since mixing to peak temperature (hr)	8	5:30	5:30
